# Supplementary material for: Molecular sensitised probe for amino acid recognition within peptide sequences
Source: Nat Commun. 2023 Dec 14;14:8335. doi: 10.1038/s41467-023-43844-5 (PMC10721870; doi:10.1038/s41467-023-43844-5)
Supplement: Supplementary file 1 — Supplementary Information [file 41467_2023_43844_MOESM1_ESM.pdf]

Supplementary information

Molecular sensitised probe for amino acid recognition within peptide sequences

Xu Wu,[1,2//] Bogdana Borca,[3,4//] Suman Sen,[1] Sebastian Koslowski,[1] Sabine Abb,[1] Daniel Pablo Rosenblatt,[1] Aurelio Gallardo,[5,6] Jesús I. Mendieta-Moreno,[5] Matyas Nachtigall,[5] Pavel Jelinek,[*,5] Stephan Rauschenbach,[*,1,7] Klaus Kern[1,8] Uta Schlickum,[*,1,3]

Affiliations:

[1]Max Planck Institute for Solid State Research, Stuttgart, Germany

[2]School of Integrated Circuits and Electronics, Beijing Institute of Technology, Beijing 100081, China

[3] Institute of Applied Physics and Laboratory for Emerging Nanometrology, Technische Universität Braunschweig, 38104 Braunschweig, Germany

[4]National Institute of Materials Physics, 077125 Magurele, Romania

[5]Institute of Physics of the Czech Academy of Science, Prague, Czech Republic

[6]Department of Condensed Matter Physics, Faculty of Mathematics and Physics, Charles University, Prague, Czech Republic

[7]Department of Chemistry, University of Oxford, Oxford, UK

[8]Institut de Physique, École Polytechnique Fédérale de Lausanne, Lausanne, Switzerland

Supplementary Results

Sensitiser

The unambiguous identification of a specific amino acid, the tryptophan W, in different predefined peptides sequences is possible in STM by an enhanced tunnelling conductance procedure when using functionalized STM tips with a sensitiser molecule, based on a preferential interaction between the sensitiser and the corresponding amino acid in the sequence. The sensitiser is a thermally derived fragment R* of the arginine amino acid,[1] obtained upon sublimation (Supplementary Figure 1) that is used for the deposition of the fragment on the Au(111) surface. For a temperature above 185ºC, the arginine (R) amino acid decomposes into the final molecule R* and NH_3, H_2O, CO_2.[1]

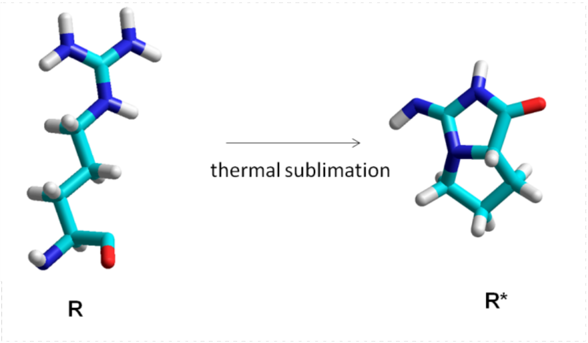

Supplementary Figure 1. Structural model of the sensitiser molecule. The sensitiser R* molecule is a derivative of the arginine (R) amino acid, resulted by thermal sublimation.

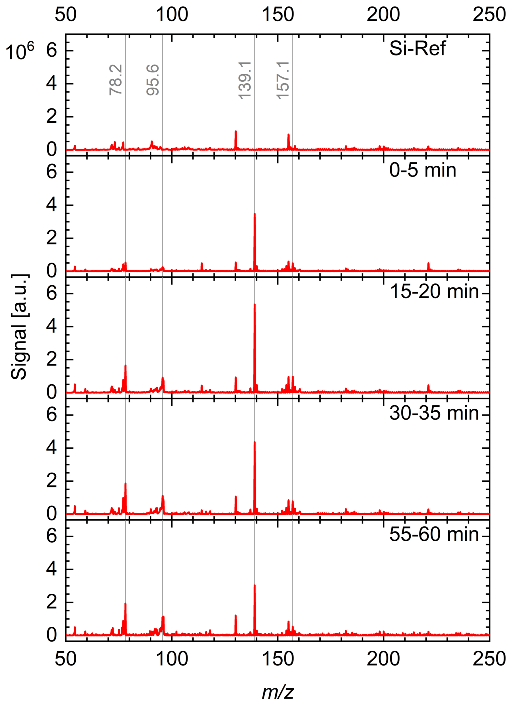

Supplementary Figure 2. Mass spectra of the sensitiser. The spectra are acquired on the Si substrate for reference and on a film of R* fragment.

Supplementary Figure 2 shows the evolution of the mass spectra acquired during the deposition of R* molecules resulted by sublimation of arginine at 195 ºC, in rapport with a reference curve acquired with the bare substrate. Excluding the features identified on the reference spectra and the other molecular ionization states, a negligible trait at *m/z* 157 may be associated to the precursor[1] of the R* fragment. The main pronounced peak characteristic of the R* fragment appears at the mass *m/z* of 139. For sensitisation, the R* molecules are deposited on the Au(111) substrate already containing the peptides. The R* molecules can be identified in the STM topographic images as a characteristic feature (Supplementary Figure 3 A) and by STS spectroscopy showing characteristic *dI/dV* features as shown in a representative spectrum in Supplementary Figure 3 C. The R* sensitiser is picked up to the tip apex following a vertical manipulation procedure that relies on switching the current feedback off and approaching the tip towards the R* molecule at a

bias voltage of about – 1 V. By rescanning the same area (Supplementary Figure 3 B), the R* does not appear as previously on the image and the aspect of the STS curve acquired with the sensitised tip on the clean Au(111) surface is distinctive in comparison with spectra measured with a metal tip (Supplementary Figure 3 C). The STS curves acquired on different sites of the molecules show a signal enhancement only on the parts corresponding to the W-W amino acids (Supplementary Figure 4).

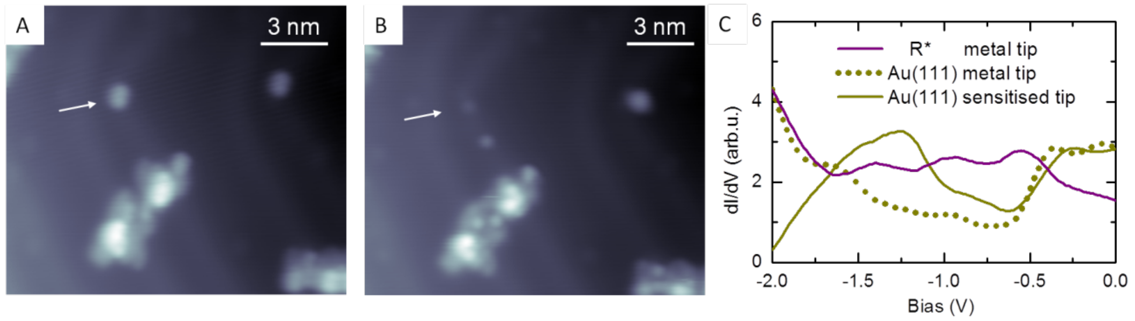

Supplementary Figure 3. STM tip sensitisation. A STM image of the R* sensitiser (marked with an arrow) in the vicinity of a sequence **1** (WW-PPPP-RR) dimer. **B** STM image in the same area as (**A**) after tip sensitisation. **C** STS characteristic curves of the R* and the clean Au(111) surface acquired with a metal and a sensitised tip, respectively.

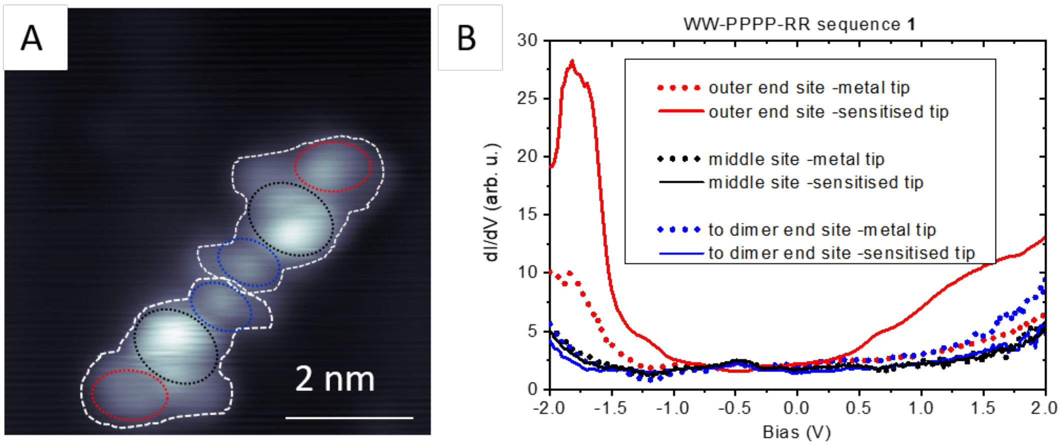

Supplementary Figure 4. STS acquired with metal and sensitised tip. A STM image of the sequence **1** (WW-PPPP-RR) dimer. **B** STS characteristic curves acquired on different sites of the molecules with a sensitized and a metal tip on the regions marked in panel (**A**).

The R* molecule may have a tautomeric configuration. Our calculations show (Supplementary Figure 5) that after the attachment of the sensitiser to the tip, the other possible tautomer is energetically much higher in energy by about 0.73 eV. Therefore, we can conclude that the tautomerization process is quenched after the tip functionalization with the tautomeric form presented in Supplementary Figure 5 B.

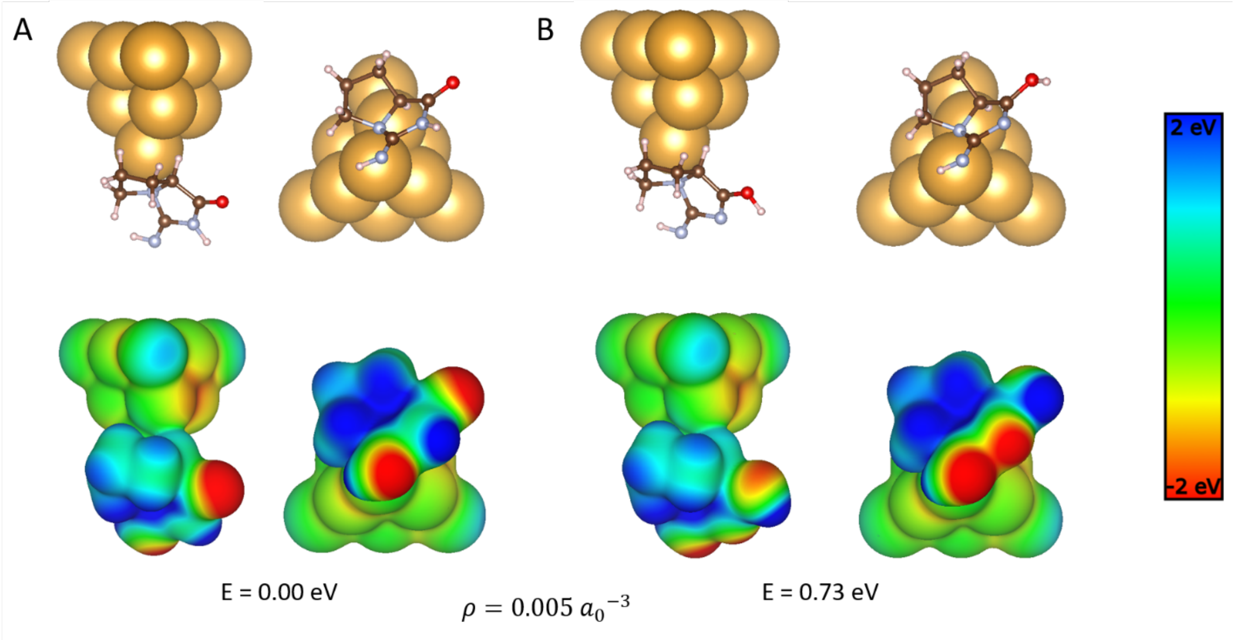

Supplementary Figure 5. Relaxed structure, EPS and total energy of the two considered tautomers of the sensitiser. A The structure presented in the main text in Fig. 2, that presents a polar character. **B** Tautomeric configuration for comparison.

The sensitiser R* molecules may adsorb on different conformations at the apex of the STM tip and it represents a fundamental aspect of sensitisation. We performed DFT calculations to find the optimal adsorption conformation of the R* unit at the tip apex. According to our DFT calculations, we found two dominant configurations with significantly lower biding energy than the other configurations. These two configurations (Supplementary Figure 6) have very similar energies, with a difference of about 0.03 eV. However, according to the DFT calculation, the second conformation of the sensitiser molecule (Supplementary Figure 6 B) does not provide adequate sensibility for recognition. This finding is consistent with experimental observations, where far from all molecules were adequate for sensitisation.

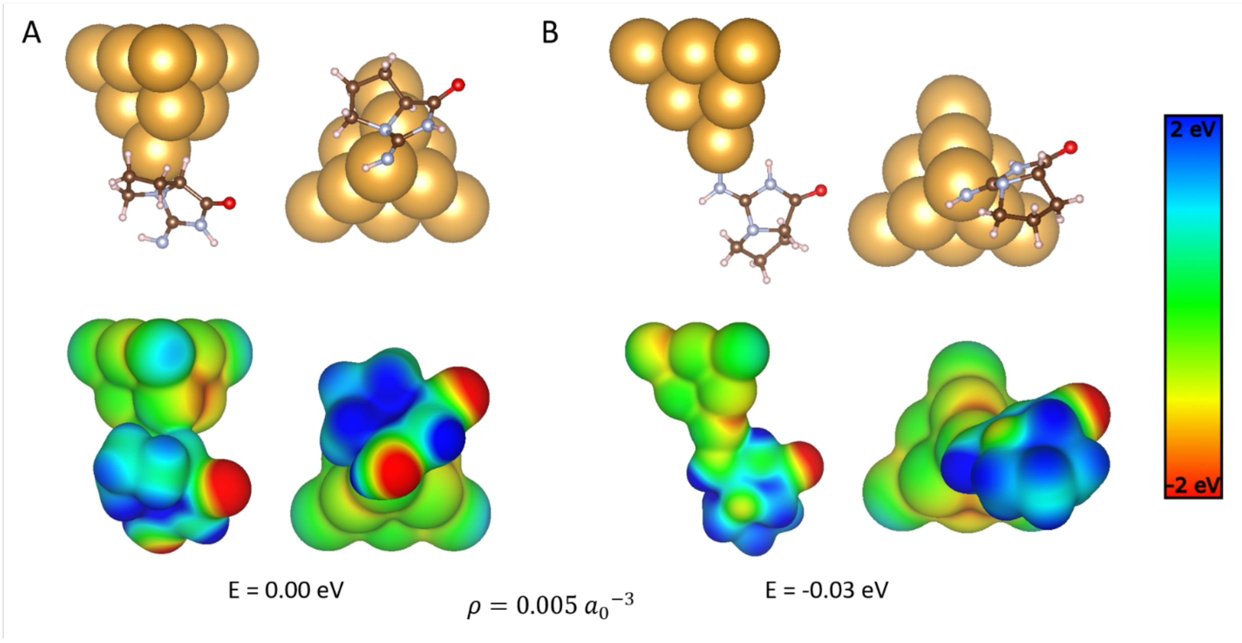

Supplementary Figure 6. Relaxed structure, EPS and total energy of the two considered configurations of the sensitiser. A The structure presented in the main text in Fig. 2, that presents a polar character. **B** An alternative configuration with similar energy that does not present the necessary polar character to achieve electrostatic selectivity.

Molecular orbital

The energy levels and molecular orbitals of the sequence **1** (WW-PPPP-RR) were calculated in the gas phase using the Density Functional Theory (DFT), with the coordinates of the peptide absorbed on the surface and relaxed with a quantum mechanics/molecular mechanism formalism (Supplementary Figure 7). The highest occupied molecular orbital (HOMO) and the next HOMO-1 states are mainly localized on the WW residue of the sequence **1**, and are responsible for the feature observed in the *dI/dV* STS spectra that allows the identification of the W amino acids.

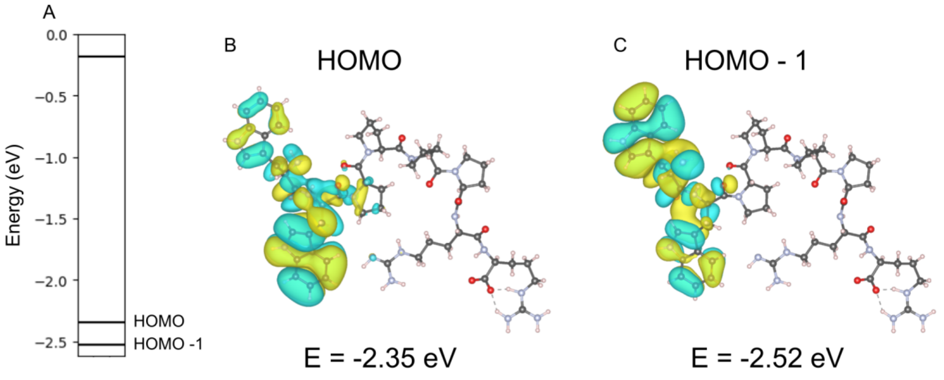

Supplementary Figure 7. Molecular orbitals of sequence 1. A Calculated energy levels of sequence **1**. **B**,**C** Spatial distribution of the HOMO (**B**) and HOMO-1 (**C**) orbitals across the peptide.

Structural Model

For the proposition of the structural model we carried out theoretical calculations for the optimization of the peptides on the surface including preliminary annealing at 300 K with subsequent relaxation. The resulting structure for the sequence **1** (WW-PPPP-RR) is represented in Supplementary Figure 8, overlapping the molecular structure (Supplementary Figure 8 A) on the STM image of the dimer (Supplementary Figure 8 B). The edge ending site (1) corresponds to the WW motif, the highest topographic feature (2) identified at the center of the peptide corresponds to the PPPP amino acids and the inner end of the peptide (3) to the RR group and the C-terminal. The calculated model fits very well the position of the AA determined experimentally (Supplementary Figure 8 C-E). The spectrocopic enhanced feature in the *dI/dV* map (Supplementary Figure 8 D) indicates the position of the WW motif within the peptide.

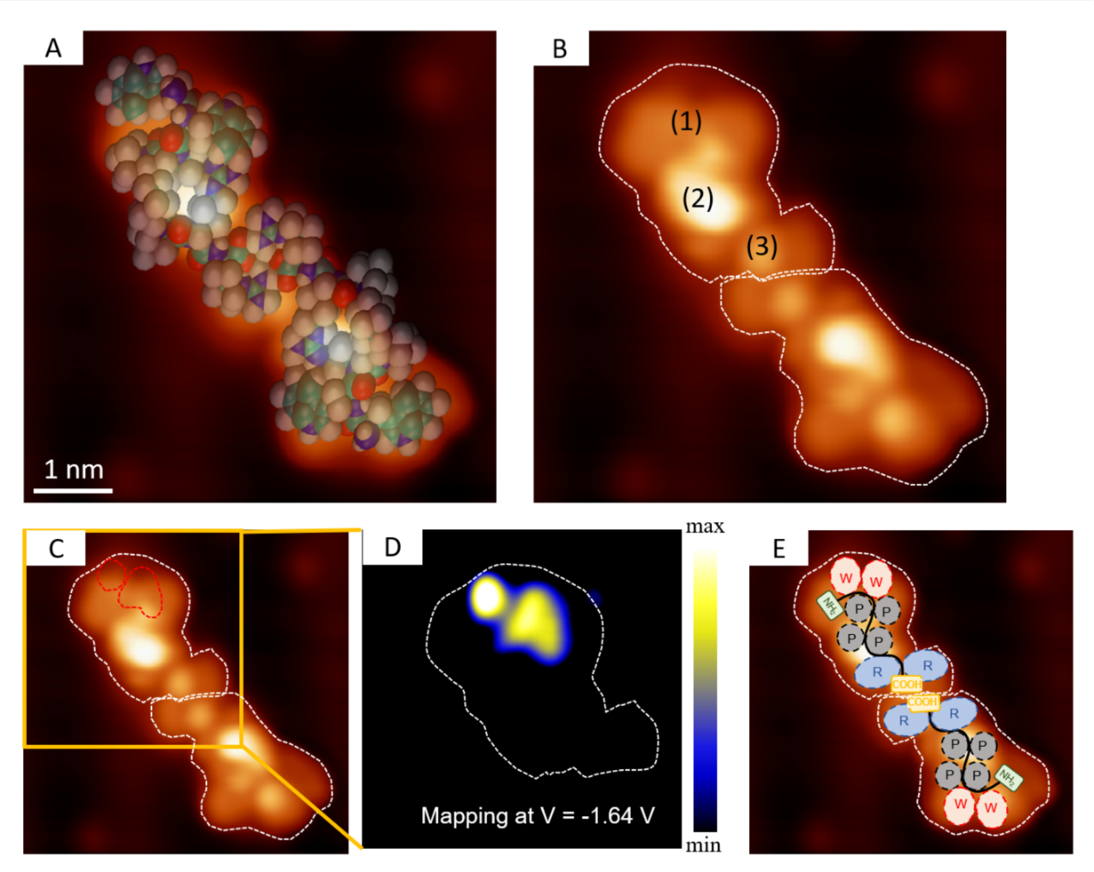

Supplementary Figure 8. Identification of the structural model of sequence 1.A Structural model calculations of the relaxed dimer of peptide **1** (WW-PPPP-RR) configuration on the surface overlapped over the STM topography. **B** STM image of the dimer of peptide **1,** marking the molecules and different parts of the peptide with (1) the edge ending, (2) the high part on the center and (3) the inner end of the molecule. **C** STM image of the dimer of peptide **1,** indicating the monomer and the feature captured in panel (**D**). **D** Spectroscopic *dI/dV* map acquired with a sensitised tip at a characteristic bias voltage of -1.64 V, that shows a significant feature enhancement towards the marked outer-end-site of the peptide.

Electrospray ion beam deposition

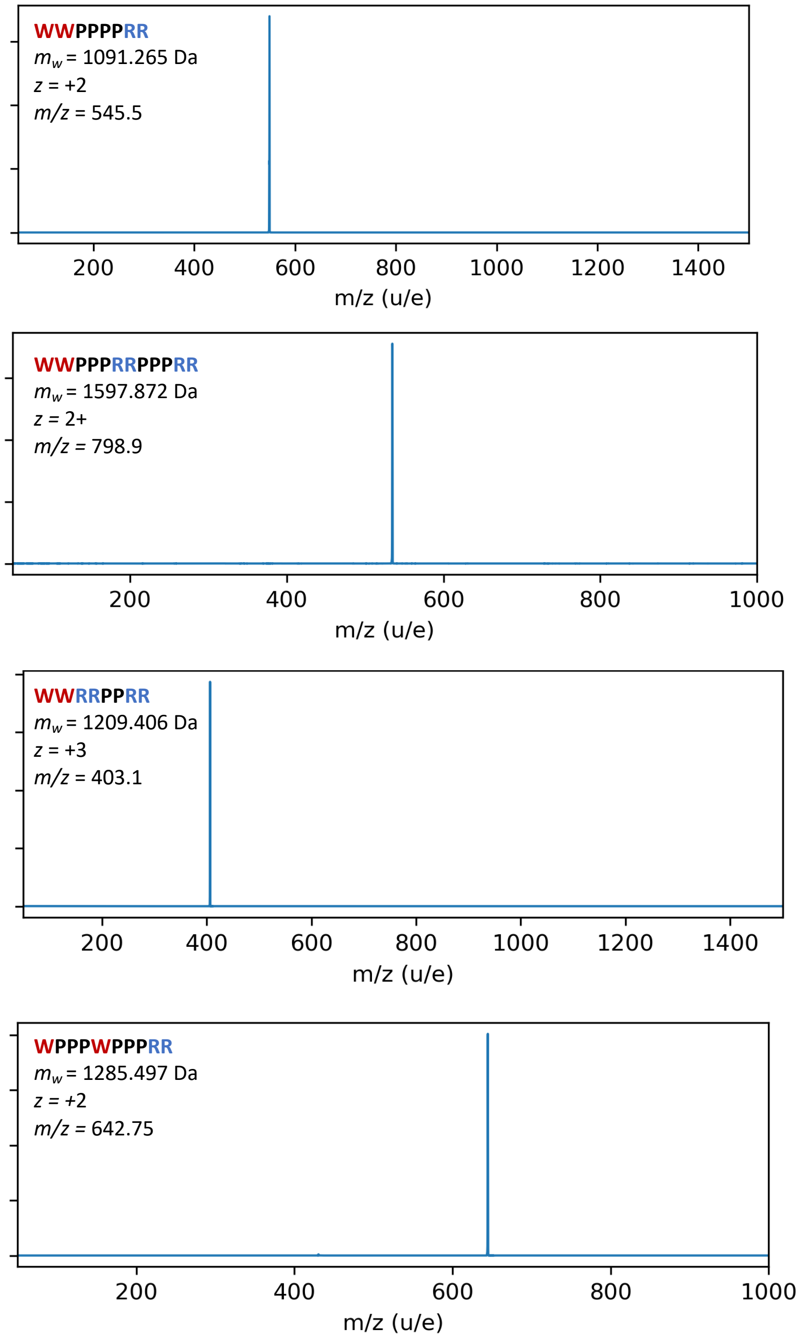

Supplementary Figure 9. Mass sepctra of the deposited sequences. Time-of-flight mass spectra of the ion beams used for deposition, recorded after mass selection.

Supplementary References.

(1) Weiss, I. M., Muth, C., Drumm, R. & Kirchner, H. O. K. Thermal decomposition of the amino acids glycine, cysteine, aspartic acid, asparagine, glutamic acid, glutamine, arginine and histidine. *BMC Biophysics* **11**:2 (2018). https://doi.org/10.1186/s13628-018-0042-4
